# The Boston Medical and Surgical Journal

**Published:** 1875-06-15

**Authors:** 


THE BOSTON MEDICAL AND SURGICAL JOURNAL.—A centennial number. The *Boston Medical and Surgical Journal* for June 17th, will be largely devoted to historical matters of special professional interest. Among the attractive features will be the following: 1, A copper-plate portrait of Gen. Joseph Warren, M.D., with quotations from his medical day book; 2, An original Sonnet by Dr. Oliver Wendell Holmes; 3, A Paper by Dr. George B. Loring, on the Medical Profession of Massachusetts at the time of the Revolution; 4, A Paper by Dr. J. M. Toner, of Washington, D. C., on the Medical Department of the Continental Army; 5, A Translation of a Hessian Surgeon's Notes of some of his American experiences; 6, Reminiscences of a Tory Surgeon's Part in the Battle of Bunker Hill; 7, A Letter from Charleston, S. C., upon Historical Subjects. Price 15 cents. H. O. Houghton and Company.

